# The Abscopal Effect Exists in Non-small Cell Lung Cancer: A Case Report and Review of the Literature

**DOI:** 10.7759/cureus.4118

**Published:** 2019-02-22

**Authors:** Jacob Bitran

**Affiliations:** 1 Oncology, Advocate Lutheran General Hospital, Park Ridge, USA

**Keywords:** abscopal effect, radiation treatment, nivolumab, non-small cell lung cancer, ipilimumab

## Abstract

The case of a 62-year-old woman who presented with stage IV adenocarcinoma of the left lung and a large left adrenal metastasis is presented. Following local radiation therapy to the left lung and nivolumab, she is now disease free 4.5 years from the time of the diagnosis. Five additional cases that have been reported are reviewed. The abscopal effect exists in non-small cell lung cancer (NSCLC) and has been reported with local radiation therapy alone as well as local radiation followed by either nivolumab or ipilimumab. Future randomized studies should address the synergist effects of local radiation therapy and the programmed cell death 1 (PD-1) checkpoint inhibitors versus the PD-1 checkpoint inhibitors alone.

## Introduction

The abscopal effect was described 50 years ago and is a phenomenon in which local radiation therapy is associated with regression of metastatic cancer at a distance from the irradiated site [1-2]. While the conventional wisdom is that locally directed ionizing radiation therapy (RT) induces lethal chromosomal changes and simultaneously activates deoxyribonucleic acid (DNA) damage response pathways [3-4], it is also at the same time immunomodulatory as RT activates nuclear factor-kappa B [5]. Garnett et al. reported that radiation therapy can alter the immuno-phenotype of cancer [6]. These authors conducted in vitro experiments with human cancer cell lines treated with sublethal radiation therapy and reported upregulation of Fas, intercellular adhesion molecule-1, mucin-1, carcinoembryonic antigen, and major histocompatibility class 1 antigens [6]. Deng and colleagues have reported that ablative radiation activates an immunologic response in that DNA from which irradiated tumor is taken up by antigen presenting cells and in turn induces type 1 interferon production [7]. These experiments support the hypothesis that alterations of the immuno-phenotype may make cancer more immunogenic and cause destruction by activating the person’s own immune system.

Pembrolizumab and nivolumab are programmed cell death 1 (PD-1) checkpoint inhibitors that are in clinical use to treat a variety of disseminated or locally advanced cancers including non-small cell lung cancer (NSCLC). These drugs are efficacious and relatively well tolerated. Adverse effects include skin rashes, hypothyroidism, autoimmune colitis, pneumonitis, and endocrinopathies including autoimmune adrenal insufficiency and hypopituitarism.

Ribeiro Gomes et al. [8] published a small study analyzing the abscopal effect coupled with anti-PD 1 therapy in a variety of patients with metastatic solid tumors. They reported the occurrence of an abscopal effect in patients with melanoma but the absence of the effect in patients with renal cell carcinoma or NSCLC.

Herein, the author reports the case of a patient with adenocarcinoma of the left lung, stage IV at presentation, who had an abscopal effect augmented by nivolumab and who is now alive and clinically disease-free 4.5 years since the time of diagnosis. In this case report, the author reviews the patients reported to date with NSCLC and an abscopal effect [9-13].

## Case presentation

**Figure 1:**
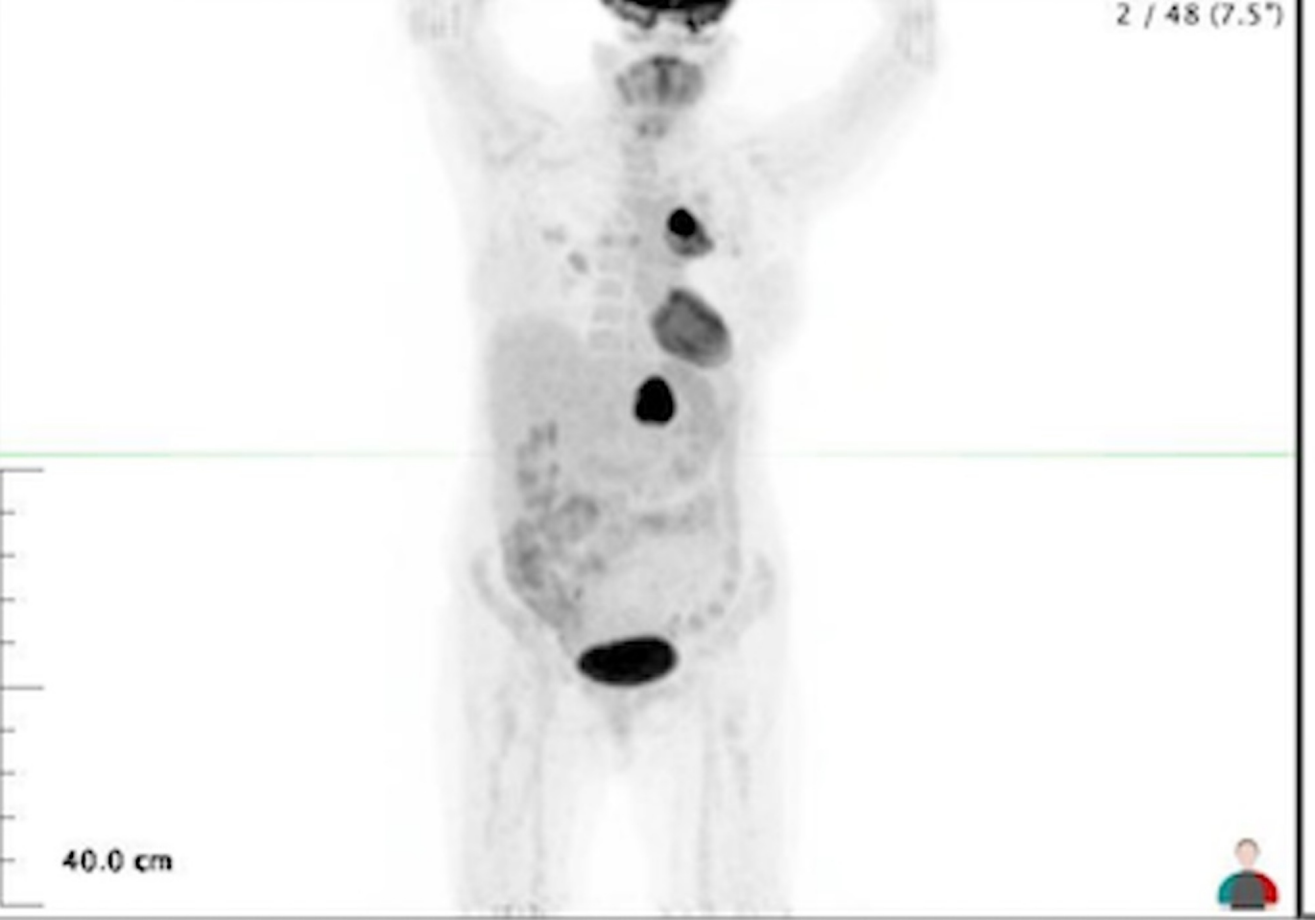
Positron emission tomography (PET) scan dated 08/07/2014 prior to radiation therapy The positron emission tomography (PET) scan shows intense uptake in the left lung and the left adrenal mass. The standardized uptake value (SUV) in the lung was 15 and SUV in the adrenal mass was 22.

A now 62-year-old woman presented to the Advocate Lutheran General Hospital (ALGH) on June 6, 2014 because of a three-week history of hemoptysis. She had a 40-pack year history of cigarette smoking. A computed tomographic (CT) scan of the chest, abdomen, and pelvis documented a 5 cm, necrotic, left lower lung mass with pathologic (3-4 cm) hilar and left mediastinal adenopathy. There was an 8 cm mass found in the left adrenal consistent with metastatic involvement. No other metastatic sites were identified. A CT guided fine needle aspiration of the adrenal mass document a poorly differentiated adenocarcinoma consistent with a primary lung adenocarcinoma based on histochemical staining (TTF-1 positive). The epidermal growth factor receptor (EGFR) and anaplastic lymphoma kinase (ALK) studies were negative. A staging magnetic resonance imaging (MRI) of the brain was normal. On her first visit, she had blood loss anemia (hemoglobin 6.9 gms/dL) because of on-going hemoptysis that she estimated to be a cupful daily. On July 1, 2014 she was hospitalized at ALGH, transfused, and started on chemotherapy with carboplatin and pemetrexed. She was discharged and seen weekly. The hemoptysis persisted, and she required one unit of packed red blood cells weekly to maintain a hemoglobin of greater than 7 gms/dL. On July 22, 2014, she received her second cycle of carboplatin and pemetrexed. She continued to have ongoing hemoptysis with weekly transfusion requirements. Following the second cycle of carboplatin and pemetrexed, a restaging chest and abdominal CT documented progressive disease as did the positron emission tomography (PET) scan (Figure 1).

The adrenal mass was now 10 cm and the lung primary was now 7 cm. She was referred to radiation oncology in an attempt to better control the ongoing hemoptysis so that she didn’t exsanguinate. She received 27 Gy to the left lower lobe lung mass in 9 fractions.

The hemoptysis started to abate and she was enrolled on a compassionate use nivolumab study, ALGH 1408, and was started on nivolumab 3 mg/kg every two weeks commencing on September 24, 2014; the patient continues on nivolumab now 480 mg monthly.

**Figure 2:**
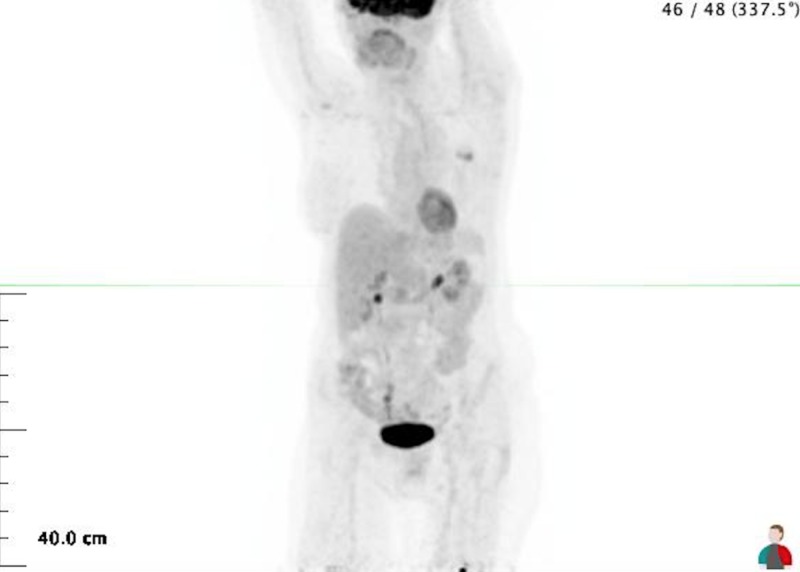
Positron emission tomography (PET) scan dated 01/25/2015 after 15 cycles of nivolumab There is no longer any uptake in the left lung.The left adrenal mass is now 2.2 cm and the SUV is 2.2.

The PET scan performed on January 25, 2015, after 15 cycles of nivolumab is shown in Figure 2. The left adrenal mass reading was an SUV of 2.2 and the size decreased to 2.2 cm.

## Discussion

There have been six case reports (including the current one) of an abscopal effect in patients with NSCLC [9-13] as shown in Table 1.

**Table 1:** Case reports of an abscopal effect in NSCLC NSCLC: non-small cell lung cancer; C&P: carboplatin and pemetrexate; V&G: vinorelbine and gemcitibine; SBRT: stereotactic body radiation therapy; RPN: retroperitoneal lymph nodes; nivo: nivolumab; ipili: ipililimumab; LUL: left upper lobe; RLL: right lower lobe.

Reference No.	Age/Sex	Stage	Prior Therapy	Radiation Therapy	Abscopal Effect
[9]	64/M	IV	C&P V&G	30Gy/5 to liver + ipili	Regression at distant sites
[10]	63/M	IV	none	45Gy/15 to CNS and 30Gy/10 to L3	Regression of lung primary
[11]	78/F	IIIA	none	60Gy/30 to LUL SBRT to RLL	Developed adrenal & humeral mets that disappeared
[12]	47/M	IIIB	Chemotherapy Radiation+ Cetuximab Resection	SBRT to 2 of 3 RPN + nivo	3rd RPN disappeared
[13]	47/M	IIIA	Chemotherapy then resection	40Gy/20 to liver + nivo	Lung mets regressed
Present Case	62/F	IV	C&P	27Gy/9 to left lung +nivo	Adrenal met disappeared

In two of the six cases [10-11], radiation therapy alone to either the primary lung cancer or a metastatic lesion leads to regression and represent a bona fide abscopal effect. Of these two patients, one had a short-lived abscopal effect [10], the other patient reported [11] had a long-lived response. In the remaining four patients [9,12-13], including the current case, the abscopal effect was augmented by either ipilimumab [9] or nivolumab [12-13]. One could certainly argue that the effect seen in these patients was not a true abscopal effect but rather the consequence of the immunotherapy used. The activity of nivolumab as a second line therapy in patients with previously treated NSCLC is modest at best. There was 17.1% overall response rate and only one complete response (CR) among 74 patients with a non-squamous histology (CR rate 1.3%) [14]. Alternatively, the counter-argument is that the local radiation therapy caused an alteration of the immuno-phenotype of the lung cancer making it more immunogenic and the immunotherapy augmented the abscopal effect. The patient reported here is alive and clinically disease-free 4.5 years after the time of initial diagnosis.

## Conclusions

In conclusion, there appears to be a bona fide abscopal effect in patients with NSCLC. The author is of the opinion that the PD-1 inhibitors augment this effect. Ultimately, the way to conclude as to whether local radiation therapy heightens the immunogenicity of NSCLC is to perform randomized studies comparing anti-PD1 therapy alone to anti-PD 1 coupled with initial local stereotactic radiation therapy to either a primary or metastatic target. Such a study will quantify whether there is an additive effect of local radiation therapy on the control of metastatic NSCLC and its impact on progression free and overall survival.
